# Serial cardiac magnetic resonance imaging for guidance of therapy management in patients treated with anakinra due to recurrent pericarditis

**DOI:** 10.1093/ehjimp/qyae019

**Published:** 2024-03-28

**Authors:** Francesco Bianco, Valentina Bucciarelli, Francesca Coretti, Serena Cataldi, Francesca Damadei, Elena Raffaelli, Nicolò Schicchi, Alessia Omenetti, Bianca Lattanzi, Emanuela Berton, Francesca Chiara Surace, Alessandra Baldinelli, Luciana Breda, Salvatore Cazzato, Carlo Catassi, Antonio Dello Russo, Sabina Gallina

**Affiliations:** Department of Pediatric and Congenital Cardiology and Cardiac Surgery, Azienda Ospedaliero Universitaria ‘Ospedali Riuniti’, Via Conca, 71, Ancona 60123, Italy; Department of Pediatric and Congenital Cardiology and Cardiac Surgery, Azienda Ospedaliero Universitaria ‘Ospedali Riuniti’, Via Conca, 71, Ancona 60123, Italy; Cardiology and Arrhythmology Clinic, University Hospital ‘Umberto I-Lancisi-Salesi’, Marche Polytechnic University, Ancona, Italy; Department of Pediatrics, Marche Polytechnic University of Ancona, Ancona, Italy; Department of Pediatrics, Marche Polytechnic University of Ancona, Ancona, Italy; Department of Pediatric and Congenital Cardiology and Cardiac Surgery, Azienda Ospedaliero Universitaria ‘Ospedali Riuniti’, Via Conca, 71, Ancona 60123, Italy; Radiology Department, Azienda Ospedaliero Universitaria ‘Ospedali Riuniti’, Ancona, Italy; Pediatric Unit, Department of Mother and Child Health, Salesi Children's Hospital, Ancona, Italy; Pediatric Unit, Department of Mother and Child Health, Salesi Children's Hospital, Ancona, Italy; Department of Pediatric and Congenital Cardiology and Cardiac Surgery, Azienda Ospedaliero Universitaria ‘Ospedali Riuniti’, Via Conca, 71, Ancona 60123, Italy; Department of Pediatric and Congenital Cardiology and Cardiac Surgery, Azienda Ospedaliero Universitaria ‘Ospedali Riuniti’, Via Conca, 71, Ancona 60123, Italy; Department of Pediatric and Congenital Cardiology and Cardiac Surgery, Azienda Ospedaliero Universitaria ‘Ospedali Riuniti’, Via Conca, 71, Ancona 60123, Italy; Department of Pediatrics, University of Chieti, Chieti, Italy; Pediatric Unit, Department of Mother and Child Health, Salesi Children's Hospital, Ancona, Italy; Department of Pediatrics, Marche Polytechnic University of Ancona, Ancona, Italy; Cardiology and Arrhythmology Clinic, University Hospital ‘Umberto I-Lancisi-Salesi’, Marche Polytechnic University, Ancona, Italy; Department of Neurosciences, Imaging and Clinical Sciences, Gabriele d’Annunzio University of Chieti-Pescara, Chieti, Italy

**Keywords:** recurrent pericarditis, anakinra, cardiac magnetic resonance, late gadolinium enhancement, C-reactive protein

## Abstract

**Aims:**

To determine the utility of serial cardiac magnetic resonance (CMR) imaging for guidance of therapy management in patients treated with anakinra due to recurrent pericarditis (RP), compared with C-reactive protein (CRP) assay alone.

**Methods and results:**

In 2018–21, we enrolled 18 (14.5 ± 1.8 years old, 72% males) consecutive RP patients treated with anakinra (100 mg/day in patients ≥ 18 years old; 2 mg/kg/day < 18 years old) due to RP corticosteroid-dependent or not responsive to colchicine or non-steroidal anti-inflammatory drugs. After hospitalization, they were 1:1 randomized to CMR [no pericardial oedema and/or late gadolinium enchantment (LGE)] or CRP (<0.6 mg/dL). Tests were repeated every 3 months until negative to halve the anakinra dosage and cessation. The idiopathic aetiology was the most prevalent (*n* = 8, 44%), followed by post-pericardiotomy (*n* = 6, 33%). After a median treatment period of 8.7 ± 3.6 months, CRP-guided RP patients experienced more recurrences than CMR-guided ones (6 vs. 1, *P* = 0.016), with the worst prognosis in terms of recurrences (log-rank, *P* = 0.025) and significantly increased time of treatment (12.7 ± 2 vs. 16.1 ± 3.4 months, *P* = 0.019). In a multivariable exploratory Cox regression model, the number of previous recurrences and the idiopathic aetiology were independent predictors of RP during the anakinra treatment. New recurrences were subsequently directed to CMR imaging, and therapy was modified according to the LGE/oedema trend. After 1-year follow-up, no further recurrence was detected.

**Conclusion:**

Among patients with RP and treated with anakinra, serial CMR imaging of the pericardium can be utilized as an imaging biomarker, more informative for therapy duration than the solely CRP assessment.

**ClinicalTrials.gov Identifier:**

NCT06071156

## Graphical Abstract

**Graphical Abstract:**
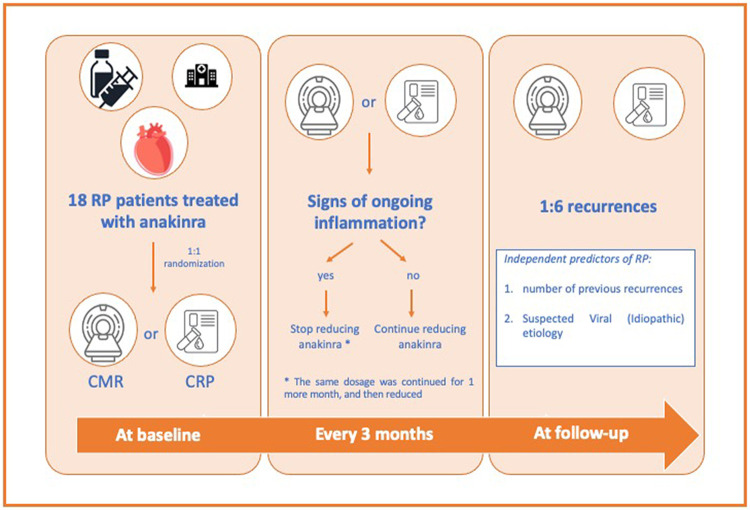

Recurrent pericarditis (RP) is a specific pathology of the pericardium included within the pericardial syndromes by the guidelines of the European Society of Cardiology (ESC). The latter defines RP as pericarditis occurring after a symptom-free interval of 4–6 weeks from a documented first episode of acute pericarditis; the recurrence rate may range from 15% to 30%, with a significant increment of 50% in patients treated with corticosteroids or not treated with colchicine. The diagnosis of recurrences follows the same criteria utilized for acute pericarditis, and a viral aetiology can often be demonstrated.^1^

The pathogenesis of RP is still debated, but they are self-sustained by an autoinflammatory/autoimmune amplified response following an exogenous or endogenous trigger. In this context, the cytokine interleukin 1 (IL-1) has been implicated as a key mediator of RP. Anakinra, an IL-1 antagonist, is of particular interest because it limits the self-sustained pathway of RP and may reduce the recurrences. The current 2015 ESC guidelines for the diagnosis and management of pericardial diseases recommend anakinra in cases of proven infection-negative, corticosteroid-dependent RP not responsive to colchicine, but it remains debated the duration of the therapy and when to start its tapering. In this context, cardiac magnetic resonance (CMR) imaging has recently emerged as an interesting imaging biomarker capable of detecting pericardial inflammation, proving pericardial oedema and late gadolinium enhancement (LGE), and distinguishing three defined pericardial inflammation phases: acute (oedema and LGE), subacute (only LGE), and burned-out (no oedema nor LGE) (*Figure 1*).^1–3^

**Figure 1:**
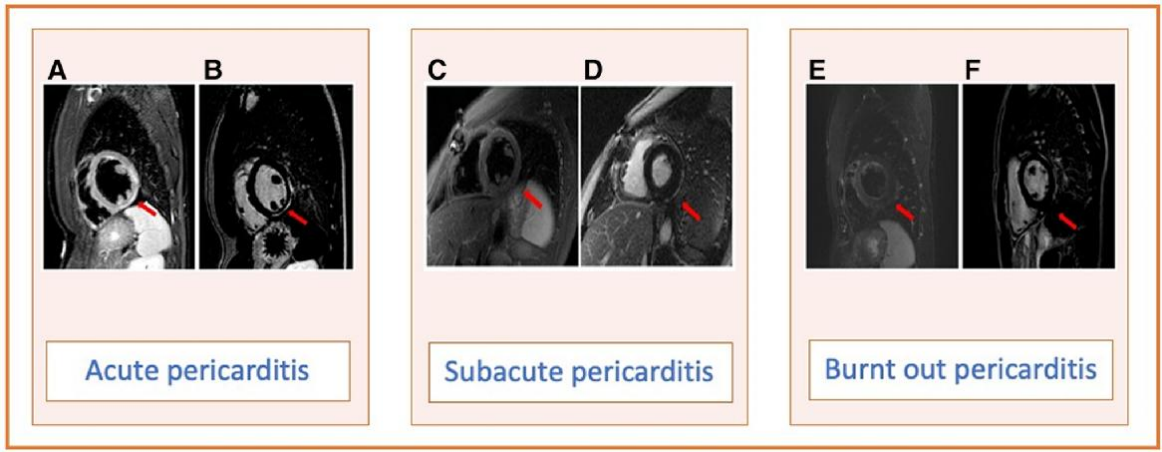
Pericardial inflammation phases: acute with oedema (*A*) and LGE (*B*); subacute with no oedema (*C*) and only LGE (*D*); burned-out with no oedema (*E*) and no LGE (*F*). The arrows indicate the inferolateral localization of pericardial inflammation over the three pericardial inflammation phases.

To overcome the 2015 ESC guidelines limitations, we sought to determine the utility of serial CMR imaging for guidance of therapy management in patients treated with anakinra due to RP, compared with the C-reactive protein (CRP) assay alone, as currently recommended.

In 2018–21, we enrolled at ‘Ospedali Riuniti’ of Ancona and ‘SS. Annunziata’ of Chieti, both in Italy, *n* = 18 (14.5 ± 1.8 years old, 72% males) consecutive patients treated with anakinra (100 mg/day if ≥18 years old and 2 mg/kg/day if <18 years old) due to corticosteroid-dependent or not responsive to colchicine or non-steroidal anti-inflammatory drugs (NSAIDs) RP. The scheme of treatment was 3 months at full dosage, the next 3 months of treatment at full dosage every other day, and the last 3 months at halved dosage every other day until the end of therapy.

Clinical assessments, laboratory tests, electrocardiogram (ECG), and echocardiography were achieved in all patients at the time of the enrolment. Next, they were 1:1 randomized to CMR [no pericardial oedema and/or late gadolinium enchantment (LGE)] or CRP (<0.6 mg/dL) (*Figure 2*). The latter guided each anakinra dose reduction following the above-mentioned therapy scheme. If the tests were found positive for ongoing pericardial inflammation, or supposed in the case of CRP, the reduction was postponed, and 1 more month of therapy was administered before the reduction.^4,5^

**Figure 2:**
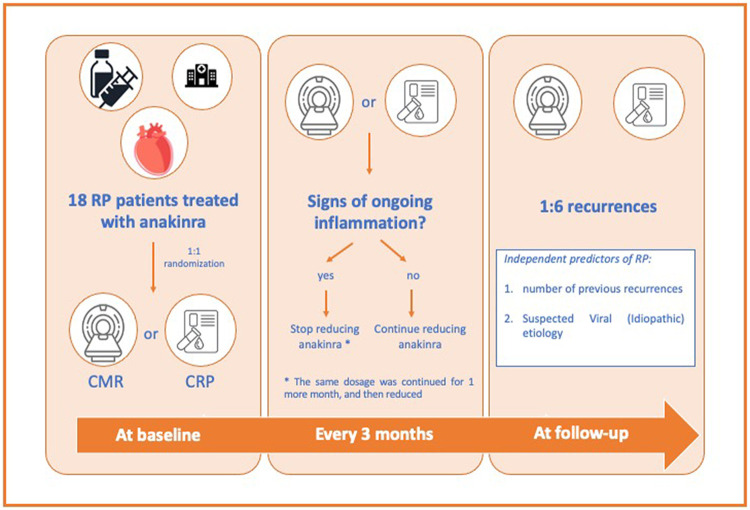
Study flow chart, from the randomization to the follow-up.

Previous medical treatment for pericarditis (corticosteroids, colchicine, or NSAIDs) was contextually administered during the whole full-anakinra treatment and suspended 1 week after halving anakinra. After anakinra cessation, patients were then followed up every 3 months for the next year with clinical assessments, laboratory tests, ECG, and echocardiography. A 1-year follow-up final CMR assessment was achieved for all the participants.^4,5^

CMR exams were conducted utilizing a 1.5 T Excite scanner (GE Medical System, Milwaukee, WI, USA) or 1.5 T Achieva (Philips Medical Systems, Amsterdam, the Netherlands).

Firstly, cine steady-state free precession images and T1/T2-weighted imaging were obtained. Secondly, ∼10 min after intravenous injection of gadolinium-based contrast agent, CMR LGE images were acquired in short-axis orientation (base/mid-cavity/apex) and in long-axis (2/3/4-chamber planes) when necessary, using a phase-sensitive inversion recovery technique with an inversion time selected for optimal nulling of the myocardium. LGE extension and oedema presence/absence were qualitatively assessed utilizing a simple visual evaluation. If LGE was present, it was graded as trivial when its extension was limited to ≤50% of the cardiac circumference in one of the three levels (base/mid-cavity/apex), mild when comprising >50% of the circumference in one of the three levels, moderate when comprising >50% of the circumference in two of the three levels, and severe when comprising >50% of the cardiac circumference of all the three levels. Oedema was graded as present/absent in the same LGE territory. Long-axis two-/three-/four-chamber planes were used to confirm the LGE/oedema extension. Images were analysed using CVI42 (Circle Cardiovascular Imaging Inc, Calgary, Canada).^3,6^

Recurrences were defined as an acute onset of chest pain, pericardial rubs, and widespread ST-elevation or PR depression at the ECG accompanied by fever or the new demonstration/worsening of pre-existing pericardial effusion at the echocardiography.^1^

In agreement with the Declaration of Helsinki, all patients enrolled gave written informed consent at the time of their evaluation. For participants < 18 years old, a parent and/or the legal tutor gave informed consent. Our institutional board (Ospedali Riuniti Ancona—SOD CCPC) approved the study protocol (Ref. CCPC- 003/18). All procedures were conducted following relevant international guidelines and our institutional regulations.

Categorical variables are expressed as percentages, while continuous variables as mean and standard deviations (± SD) or median and interquartile intervals (Q1 and Q3), as appropriate. Continuous variables were compared by the Student’s *t*-test or non-parametric, while categorical variables were compared by the *χ*^2^ test or Fisher’s exact test over CMR/CRP-guided therapy management, when appropriate. Cumulative event-free survival rates over time were obtained using the Kaplan–Meier method. The log-rank test was used to compare the event-free survival curves. Utilizing a Cox proportional hazard regression analyses assessed the multivariable associations of risk covariates with recurrences. A two-tailed *P*-value of 0.05 was considered statistically significant. All statistical analyses were performed with Stata v14.1 (StataCorp, College Station, TX, USA) and Prism 8.0 (GraphPad Software, La Jolla, CA, USA).

Demographics, clinical characteristics, laboratory tests, pericarditis aetiology, medications, and a follow-up summary are presented in *Table 1*, according to the therapy management groups.

**Table 1:** Participants’ characteristics according to groups of therapy management

	Total	CMR-guided	CRP-guided	
	*n* = 18	*n* = 9	*n* = 9	*P*-value
**General characteristics**				
Age (yr.)	14.5 ± 1.8	14.0 ± 1.7	15.0 ± 1.9	0.26
Male gender, *n* (%)	13 (72%)	7 (78%)	6 (67%)	0.60
HR (bpm)	74.0 ± 10.7	73.1 ± 4.2	74.9 ± 15.0	0.74
SBP (mmHg)	119.4 ± 4.7	120.7 ± 3.8	118.1 ± 5.3	0.26
DBP (mmHg)	69.8 ± 4.2	68.2 ± 2.2	71.3 ± 5.2	0.12
**Aetiology**				
Post-pericardiotomy	6 (33%)	4 (44%)	2 (22%)	**0**.**001**
Bacterial	4 (22%)	0 (0.0%)	4 (44%)	
Suspected viral (idiopathic)	8 (44%)	5 (55%)	3 (33%)	0.059
**Medications treatment**				
Colchicine and ibuprofen	5 (28%)	4 (44%)	1 (11%)	**0**.**0001**
Ibuprofen and Indomethacin	11 (61%)	5 (55%)	6 (67%)	0.54
Aspirin	2 (11%)	0 (0.0%)	2 (22%)	
**Laboratory**				
CRP (mg/dL)	3.0 [2.0, 3.0]	3.5 [2.7, 4.5]	4.2 [2.7, 4.5]	0.96
Nadir-CRP (mg/dL)	3.9 [2.7, 4.5]	5.1 [5.0, 6.1]	6.6 [6.1, 11.5]	**0**.**030**
Hs-TnI (ng/L)	6.1 [5.1, 9.9]	4.1 [4.0, 5.8]	4.4 [4.1, 6.8]	0.44
BNP (pg/mL)	2.3 [1.6, 4.4]	45.0 [5.0, 58.0]	57.0 [45.0, 73.0]	0.69
**Follow-up**				
Number of previous recurrences	2.6 ± 0.8	2.2 ± 0.7	3.0 ± 0.9	**0**.**049**
Post-randomization recurrences	7 (39%)	1 (11%)	6 (68%)	**0**.**016**
Months of treatment	8.7 ± 3.6	12.7 ± 2	16.1 ± 3.4	**0**.**019**

Measures are reported as mean and standard deviation (± SD), number and percentage (%), or median and Q1–Q3 (interquartile interval) were appropriate.

bpm, beats per minute; BNP, brain natriuretic peptide; CMR, cardiac magnetic resonance; CRP, C-reactive protein; dL, decilitres; DBP, diastolic blood pressure; HR, hear rate; Hs-TnI, high-sensitive Troponin I; L, litres; mg, milligrams; mL, millilitres; mmHg, millimetres of mercury; ng, nanograms; %, percentage; pg, picograms; SBP, systolic blood pressure; yr, years.

The idiopathic aetiology was the most prevalent (*n* = 8, 44%), followed by post-pericardiotomy (*n* = 6, 33%). Before starting the anakinra treatment, our patients experienced a median of 2.6 ± 0.8 recurrences. Colchicine, administered with ibuprofen, was the most utilized treatment as a first-line approach substituted by ibuprofen and indomethacin at the failure of the previous treatment (*n* = 11, 61%). At the time of enrolment, all the participants presented with chest pain and the new demonstration of pericardial effusion, accompanied by CRP increment, while only *n* = 6 (33%) presented with a new demonstration of ST changes at the ECG.

Baseline CMR documented the presence of acute pericardial inflammatory phase (LGE and oedema) in the whole sample (*n* = 9, 100%), with 1 severe presentation (idiopathic aetiology), 6 moderate (*n* = 4 idiopathic and *n* = 1 post-pericardiotomy), and 3 mild (post-pericardiotomy); none was trivial. At 6 months, the majority (*n* = 7, 78%) revealed subacute pericarditis (LGE without oedema), both idiopathic. The participant with severe presentation experienced the recurrence after 6 months of treatment, during the 3-month halved dosage period, with subacute CMR demonstration.

After a median treatment period of 8.7 ± 3.6 months, CRP-guided RP patients experienced more recurrences than CMR-guided ones (6 vs. 1, *P* = 0.016), with the worst prognosis in terms of recurrences (log-rank, *P* = 0.025) and a significantly increased time of anakinra treatment (12.7 ± 2 vs. 16.1 ± 3.4 months, *P* = 0.019). In a multivariable exploratory Cox regression model, the number of previous recurrences and the idiopathic aetiology were independent predictors of RP during the anakinra treatment [hazard ratio (HR) 3.8, 95% confidence interval (CI): 1.42–10.3, *P* < 0.001, HR 1.47, 95% CI: 1.26–12.8, *P* = 0.015, respectively].

RP recurrences in the CRP-guided group were subsequently directed to CMR imaging, and therapy was modified according to the LGE/oedema trend. CRP-guided recurrences revealed at the CMR exam subacute pericarditis, with moderate extension: *n* = 3/6 idiopathic (50%), *n* = 2/6 bacterial (33%), and *n* = 1/6 post-pericardiotomy (17%). After 1-year follow-up, no further recurrences were then detected and no oedema or LGE was found at the final CMR investigation.

This study adds to previous literature supporting the concept of pericardial LGE as a potential imaging biomarker that may inform about the duration of anakinra treatment, identifying the predisposition for recurrences and avoiding any premature therapy cessation in otherwise clinically stable patients, presenting with CRP normalized. As already mentioned, the RP pathogenesis is still debated, but an autoinflammatory/autoimmune substrate, amplified by an exogenous or endogenous trigger, is the most credited one. In this context, the IL-1 probably plays a key role as a mediator of RP, in which the solely CRP assessment is not sufficient to detect the entire cessation of pericardial inflammation.^2^

Nonetheless, some study limitations should be highlighted. First, even if 1:1 is randomized, the absence of a double-blind study protocol does not fully exclude any selection bias. Second, we acknowledge *n* = 18 participants as a limitation and suggest that our findings should be interpreted as preliminary, necessitating further research with a larger sample. Third, it is documented that LGE presence can be found after cardiac surgery in asymptomatic patients without pericarditis.^7^ However, our post-pericardiotomy participants were symptomatic presenting acute pericarditis (both at CMR and CRP) and met ESC criteria at the enrolment. However, the latter cannot be currently neglected as a confounder. Fourth, the follow-up period of 1 year may not be sufficient to fully understand the long-term efficiency of this CMR-guided approach. Besides, and in accordance with all the limitations mentioned above, further study of pericardial CMR evaluation in larger patient cohorts, adjusted for clinical variables, is warranted along with its correlation with IL-1 levels and assay to reach any conclusive assumption. Finally, the increment of CMR scans needs to be investigated in a cost-effective analysis before any guideline recommendation, compared with the prospective of treatment expensiveness and hospitalizations.

In conclusion, among patients with RP and treated with anakinra, serial CMR imaging of the pericardium can be utilized as an imaging biomarker, more informative than the solely CRP assessment for therapy duration and tapering, as suggested by the current ESC guidelines.

## Data Availability

The data underlying this article are all available in the article.

## Lead author biography

Francesco Bianco, MD, PhD, FEACVI is a cardiologist and cardiovascular imager at Pediatric Cardiology and Adult Congenital Heart Disease, Azienda Ospedaliero-Universitaria ‘Ospedali Riuniti’ of Ancona, Italy. Dr Bianco earned his International PhD in Radiology with a particular focus on Cardiac Imaging from the ‘G. d’Annunzio’ University of Chieti-Pescara’ and his medical degree from the University of Pisa, Italy. Following completion of his cardiology residency at ‘G. d’Annunzio’ University of Chieti-Pescara’, he continued his post-doctoral training with a 3-year fellowship in advanced heart failure and heart transplantation and advanced cardiovascular imaging at the ‘Brigham and Women Hospital’, Boston, MA, USA, at the ‘Fondazione Toscana—Gabriele Monasterio’ Pisa, Italy, and at ‘ASST Grande Ospedale Metropolitano Niguarda’, Milano, Italy. His clinical and research interests include heart failure, congenital heart disease, and cardiac imaging.
